# Peri-Ovarian Mitochondrial Administration Improves Surrogate Markers of Ovarian Function in Aged Rats: Free Versus Alginate-Encapsulated Mesenchymal Stromal Cell-Derived Mitochondria

**DOI:** 10.3390/biomedicines14102394

**Published:** 2026-10-20

**Authors:** Zehra Seda Halbutoğulları, Zeynep Ece Utkan Korun, Gülşen Çelebi, Selenay Furat, Meryem Dişli, Gökhan Duruksu, Tijen Utkan

**Affiliations:** 1 Department of Medical Biology, Faculty of Medicine, Kocaeli University, 41001 İzmit, Kocaeli, Turkey; 2 Center for Stem Cell and Gene Therapies Research and Practice, Kocaeli University, 41001 İzmit, Kocaeli, Turkey; 3 Department of Stem Cell, Institute of Health Sciences, Kocaeli University, 41001 İzmit, Kocaeli, Turkey; 4 Department of Obstetrics and Gynecology, Faculty of Medicine, Yeditepe University, 34755 Ataşehir, Istanbul, Turkey; 5 Department of Pharmacology, Faculty of Medicine, Kocaeli University, 41001 İzmit, Kocaeli, Turkey; 6 Department of Histology and Embryology, Faculty of Medicine, Kocaeli University, 41001 İzmit, Kocaeli, Turkey

**Keywords:** ovarian aging, mitochondrial transfer, mesenchymal stromal cells, alginate, folliculogenesis, ovarian reserve

## Abstract

**Background:** Ovarian aging is driven in part by mitochondrial dysfunction, and transfer of exogenous mitochondria has been proposed as a cell-free alternative to stem cell therapy; whether the delivery format alters efficacy has not been tested directly. **Methods:** We compared free and alginate-encapsulated mitochondria isolated from rat bone marrow-derived mesenchymal stromal cells (BM-MSCs) in aged rats. Mitochondria retained their membrane potential after encapsulation; approximately 35% of the encapsulated protein was released within 1 h in vitro, and release was complete by 24 h. Thirty-five female Wistar rats were allocated to five groups (n = 7): young control (Y-Ctrl), aged control (O-Ctrl), and three aged subgroups receiving empty capsules (Alg), free mitochondria (MSC-Mito), or encapsulated mitochondria (MSC-Mito@Alg), administered peri-ovarianly and evaluated on day 14. **Results:** Both treatments were associated with higher primordial and secondary follicle numbers, fewer atretic follicles, higher estradiol, higher *Esr1*, *Fshr*, and *Sod2* expression, lower *Casp3* and *Mct4* expression, and lower Caspase-3 and higher Ki-67 immunoreactivity relative to aged controls. Anti-Müllerian hormone, *Nrf2*, and *Tomm20* were higher only with MSC-Mito@Alg; corpus luteum counts were higher only with MSC-Mito. Although empty capsules were not inert, peri-ovarian mitochondrial administration improved follicular, endocrine, and molecular indices of ovarian aging. **Conclusions:** The encapsulated formulation was associated with additional effects on antioxidant and mitochondrial-mass endpoints but was not uniformly superior to free mitochondria.

## 1. Introduction

Ovarian aging is characterized by a progressive decline in reproductive capacity and endocrine function, driven by follicle depletion, cellular senescence, and mitochondrial dysfunction [1]. Because this decline begins earlier than that of most other organ systems, the ovary offers a tractable model in which age-related mitochondrial failure and its reversal can be examined within a short experimental window. Current interventions address the consequences rather than the underlying cellular changes: long-term hormone replacement therapy carries an increased breast cancer risk [2], cytokine-based treatments are costly and lack large-scale applicability, and herbal formulations remain insufficiently characterized. Assisted reproductive technologies, including the “freeze-all” strategy for oocytes or high-quality embryos, preserve existing gametes without modifying the aged ovarian microenvironment, and success rates in women of advanced reproductive age remain suboptimal.

Mesenchymal stromal cells (MSCs) derived from various tissues have emerged as promising candidates for ovarian rejuvenation therapy. Human amniotic fluid–, placental-, and umbilical cord–derived MSCs and their exosomes restore ovarian function, attenuate oxidative stress and apoptosis, and stimulate primordial follicle activation [3,4,5]. These effects occur predominantly through paracrine mechanisms rather than direct cell replacement, and among them the transfer of functional mitochondria to recipient cells has emerged as a distinct effector mechanism, restoring oxidative metabolism and limiting apoptosis in damaged tissues [6,7,8]. Whole-cell administration, however, carries poor retention, potential immunogenicity, and safety concerns, which has directed attention to cell-free alternatives; isolated mitochondria from menstrual blood-derived stem cells have been reported to improve the ovarian condition of aged mice [9].

Mitochondria are central regulators of ovarian physiology and stem cell function. Granulosa cells are particularly enriched in mitochondria and support oocyte development by supplying metabolic substrates and ATP [10,11]. With advancing age, mitochondrial quantity and function decline, accompanied by reduced membrane potential, impaired ATP production, and increased apoptosis [1,12], which compromises oocyte quality and diminishes ovarian reserve. Beyond energy production, mitochondria act as signaling organelles that modulate cell fate through metabolic and reactive oxygen species–mediated pathways, and mitochondrial dysfunction is closely linked to age-related loss of regenerative capacity [11,13]. Accordingly, mitochondrial mass, the NRF2-dependent antioxidant response, and the glycolytic shift that accompanies mitochondrial insufficiency provide accessible readouts of whether exogenous mitochondria act on the aged ovary through the intended mechanism.

A remaining problem is delivery. Free mitochondria injected into tissue disperse rapidly, and alginate hydrogels—biocompatible, mildly gelling, and structurally similar to extracellular matrix—have been widely used to retain cells and bioactive cargo at a target site [14,15,16], including mitochondria in a rat model of myocardial infarction [17]. Whether the delivery format alters the efficacy of isolated mitochondria has not been tested directly in the ovary. We therefore compared free and alginate-encapsulated mitochondria isolated from rat bone marrow-derived MSCs, administered peri-ovarianly in a physiological rat aging model, and assessed follicular architecture, serum hormones, and the expression of genes reporting ovarian function (*Fshr*, *Esr1*, *Amhr2*), apoptosis (*Casp3*), antioxidant defense (*Nfe2l2*/*Nrf2*, *Sod2*), metabolic stress (*Slc16a3*/*Mct4*), and mitochondrial mass (*Tomm20*).

## 2. Materials and Methods

### 2.1. Study Design

This study used thirty-five female Wistar Albino rats, aged 8 and 15 months, with approval from the Animal Experiments Local Ethics Committee of Kocaeli University (Approval No: KOÜ HADYEK 6/4-2022). Body weight at the start of the experiment was 218 ± 12.83 g for young rats and 254 ± 14.77 g for old rats. Animals were bred and housed in-house at the Kocaeli University Experimental Medicine Research and Application Unit (DETAB), where all experimental procedures were also carried out; as animals were not transported from an external source, no acclimatization period was required. All animals were experimentally naïve and had not undergone any prior procedures, and only clinically healthy females, free of visible coat or structural abnormalities, were selected for the study. Animals were housed 2–3 per cage under standard laboratory conditions, at a room temperature of 24 °C, relative humidity of 40–50%, with wood-chip bedding and a 12 h light/dark cycle, and were provided with ad libitum access to food and water throughout the study duration. Surgical procedures were performed under anesthesia induced with an intramuscular combination of ketamine (90 mg/kg) and xylazine (10 mg/kg). Postoperative analgesia was provided with a single intramuscular dose of meloxicam (0.5 mg/kg). No animals died because of the surgical or injection procedures, and no unexpected adverse events or treatment-related morbidity were observed in any group during the study period.

The control groups, comprising 8- and 15-month-old rats, received standard chow and water for two weeks without additional interventions. To minimize hormonal variability, vaginal smear cytology was conducted each morning at 9:00 h (Figure S1), and animals identified as being in the estrus phase of the estrous cycle underwent the surgical or injection procedure that afternoon. This inclusion criterion was defined a priori. No specific exclusion criteria were formally pre-specified; however, animals that developed health problems preventing continuation of the experiment or that no longer met the inclusion criteria would have been excluded.

Mitochondrial preparations were administered to the peri-ovarian region of aged rats either in free form or encapsulated within alginate hydrogels. The therapeutic effects of free and alginate-encapsulated mitochondria on ovarian aging were evaluated in comparison with alginate-only and untreated control groups. The group size was determined a priori by power analysis using G*Power 3.1, with primordial follicle count as the primary outcome; assuming a large effect size (f = 0.40), a significance level of α = 0.05, and a statistical power of 0.80, seven animals per group were required. Young animals were allocated directly to the Y-Ctrl group, as this group did not require randomization. The remaining aged animals were pooled, and group allocation among the four aged groups (O-Ctrl, Alg, MSC-Mito, and MSC-Mito@Alg) was determined by simple randomization via random selection (drawing) from this pool, without the use of a computer-generated sequence. All histological, immunohistochemical, and immunofluorescence assessments, including follicle counting and image quantification, were performed by an observer blinded to group allocation. The individual animal was treated as the experimental unit for all analyses.

The animals were randomly allocated into five experimental groups (n = 7 per group) as follows: Group 1: Young control (8 months old) with no treatment

Group 2: Old control (15 months old) with no treatment

Group 3: Old + alginate control (15 months old) receiving an empty alginate gel

Group 4: Old + free mitochondria (MSC-Mito) (15 months old) receiving isolated mitochondria obtained from bone marrow–derived MSCs

Group 5: Old + encapsulated mitochondria (MSC-Mito@Alg) (15 months old) receiving alginate-encapsulated isolated mitochondria obtained from bone marrow–derived MSCs

The individual animal was treated as the experimental unit for all analyses; for each animal, one ovary was used for qRT-PCR and the contralateral ovary for histological, immunohistochemical, and immunofluorescence analyses, but group comparisons were performed at the animal level, not the ovary level.

### 2.2. Characterization of Rat Bone Marrow-Derived Mesenchymal Stromal Cells (rBM-MSCs)

Rat bone marrow-derived mesenchymal stromal cells (rBM-MSCs) were obtained from the cell collection at the Center for Stem Cell Research and Development (KOGEM), Kocaeli University. In culture, the cells displayed the characteristic spindle-shaped fibroblastic morphology (Figure S2B). The immunophenotypic characterization of rBM-MSCs was performed by flow cytometry as previously described [18]. Briefly, cells were analyzed for the expression of mesenchymal stromal cell–associated surface markers and the absence of hematopoietic markers (Figure S2A).

Osteogenic differentiation and Alizarin Red S staining were carried out according to established protocols (Figure S2C) [18]. Adipogenic differentiation was confirmed by Oil Red O staining as previously reported (Figure S2D) [19]. Chondrogenic differentiation was conducted following previously validated procedures (Figure S2E) [18].

### 2.3. Isolation and Characterization of Mitochondria

Mitochondria were isolated from rBM-MSCs through differential centrifugation. The rBM-MSCs were suspended in 500 µL of Sucrose-HEPES-EGTA (SHE) buffer (0.25 M sucrose, 20 mM HEPES (pH 7.4), 2 mM EGTA, 10 mM KCl, 1.5 mM MgCl_2_, and 0.1% BSA) at a concentration of 2 × 10^6^ cells and homogenized 10 times on ice using an insulin syringe. The homogenate was subjected to centrifugation at 2000× *g* for 20 min at 4 °C to eliminate cell debris, and the resulting supernatant was collected. This supernatant was further centrifuged at 10,000× *g* for 20 min to obtain the mitochondrial fraction, which was then resuspended in a volume adjusted according to the intended downstream application (as specified in the relevant sections below) [6,7].

To confirm that the donor cells contained mitochondria with an intact membrane potential (ΔΨm) prior to isolation, intact rat bone marrow-derived mesenchymal stromal cells (rBM-MSCs) were stained with Tetramethylrhodamine methyl ester (TMRM, Catalog No. M20036, Thermo Fisher Scientific, Eugene, OR, USA) before the isolation procedure. The cells were washed twice with phosphate-buffered saline (PBS) and incubated with 100 nM TMRM in serum-free culture medium at 37 °C for 30 min under light-protected conditions. After staining, the cells were washed with PBS and nuclei were counterstained with DAPI. Fluorescence images were acquired under identical acquisition settings for all groups using a laser scanning confocal microscope (Leica TCS SP8, Leica Microsystems, Wetzlar, Germany) and analyzed with the LAS X software (version 1.4.7.28982, Leica Microsystems, Wetzlar, Germany) [8].

The mitochondrial preparation derived from 2 × 10^6^ cells per ovary was resuspended in 100 µL and administered to the peri-ovarian regions of each ovary [20,21]. Administration was performed under ketamine–xylazine anesthesia. The injection area was disinfected with 70% ethanol. The suspension was injected percutaneously into the peri-ovarian region of each ovary, using a 26-gauge needle syringe that was inserted slowly, guided by external anatomical landmarks and without laparotomy, so that the peri-ovarian compartment remained closed and dispersion of the liquid formulation away from the ovarian surface was limited (Figure S3).

### 2.4. Preparation and Characterization of Alginate Gel Capsules

Sodium alginate powder (Sigma-Aldrich, St. Louis, MO, USA) was dissolved in 0.9% NaCl containing HEPES to obtain a 2% (*w*/*v*) solution at ambient temperature. For mitochondrial encapsulation, mitochondrial preparations obtained from approximately 2 × 10^6^ rBM-MSCs per ovary were combined with the alginate solution under sterile conditions. The resulting alginate–mitochondrial suspension was dispensed dropwise into a 1% (*w*/*v*) calcium chloride solution to facilitate ionic crosslinking, thereby forming three-dimensional (3D) alginate capsules [9,22].

To deliver a mitochondrial dose equivalent to that used for free peri-ovarian administration (the preparation from 2 × 10^6^ cells per ovary), the mitochondrial preparation for each ovary was concentrated and normalized to a resuspension volume of 20 µL prior to encapsulation. This yielded ten capsules per ovary, that is, twenty capsules per animal. Although the total mitochondrial dose was preserved, resuspension in a reduced volume increased mitochondrial concentration and facilitated homogeneous distribution within the alginate matrix during encapsulation. The final formulation consisted of ten alginate capsules per ovary. After anesthesia with ketamine and xylazine and disinfection of the skin with 70% ethanol, a dorsal incision was made to access the ovaries via a retroperitoneal approach, and the capsules were carefully placed around each ovary in the peri-ovarian space using a sterile micro-spatula. The retroperitoneal layer was closed with 6/0 biodegradable suture, and the skin was closed with 5/0 polypropylene (Prolene) suture.

To evaluate the release profile of mitochondria-associated proteins from the alginate hydrogel, mitochondria isolated from 2 × 10^6^ rBM-MSCs were mixed with sterile sodium alginate solution at a 1:1 (*v*/*v*) ratio to prepare mitochondria-loaded alginate constructs. The constructs were immersed in 1 mL sterile phosphate-buffered saline (PBS) and incubated at 37 °C under sterile conditions. At 1, 2, 3, 12, and 24 h, the entire release medium was carefully collected and replaced with an equal volume of fresh PBS to maintain sink conditions throughout the experiment. The collected supernatants were stored at −80 °C until further analysis. The concentration of mitochondria-associated proteins released into the surrounding medium was quantified using the SMART™ BCA Protein Assay Kit (Cat. No. 21071, iNtRON Biotechnology, Seongnam, Republic of Korea) according to the manufacturer’s instructions. Blank alginate constructs prepared without mitochondria were analyzed under identical conditions, and the corresponding background protein values were subtracted from the experimental measurements to normalize the release data. The cumulative amount of released protein at each time point was calculated by summing the protein measured in the collected release media from all previous sampling intervals. The cumulative release percentage was determined by normalizing the cumulative released protein to the total protein content initially encapsulated within the alginate construct using the following equation:

Cumulative protein release (%) = (Cumulative released protein/Initial encapsulated protein) × 100

Mitochondrial membrane potential (ΔΨm) was evaluated using the JC-1 fluorescent probe. Following mitochondrial isolation, the mitochondrial suspension was divided into two equal fractions. One fraction was incubated at 56 °C for 30 min to disrupt mitochondrial membrane potential and was used as a depolarized mitochondrial control. The second fraction was maintained under the standard experimental conditions and used as the functionally active mitochondrial group. Both mitochondrial fractions were incubated with JC-1 staining solution (ABP Biosciences, Rockville, MD, USA) at 1 µg/mL for 10 min at 37 °C in the dark. Following staining, the mitochondria were washed 3 times with SHE buffer to remove unbound dye. JC-1-stained active and heat-depolarized mitochondria were separately mixed with sterile sodium alginate and encapsulated using the same procedure described above for the preparation of mitochondria-loaded alginate constructs. The alginate-encapsulated mitochondria were examined using confocal laser scanning microscopy. JC-1 monomers, indicative of low mitochondrial membrane potential, were detected in the green fluorescence channel, whereas JC-1 aggregates formed in polarized mitochondria were detected in the red fluorescence channel. Images were acquired using identical laser intensity, detector gain, exposure, and image-processing settings for the active and heat-depolarized mitochondrial groups. Mitochondrial membrane polarization was qualitatively evaluated by comparing the relative green and red fluorescence signals within the alginate constructs. Blank alginate capsules prepared without mitochondria were also stained with JC-1 and imaged under identical conditions; no green or red fluorescence signal was detected in these constructs, confirming the absence of background autofluorescence from the alginate matrix itself.

### 2.5. Tissue Collection

On day 14 after treatment administration, blood samples were collected by cardiac puncture from anesthetized rats, after which the animals were euthanized. One ovary from each animal was collected for quantitative real-time polymerase chain reaction (qRT-PCR) analysis, whereas the contralateral ovary was processed for histological, immunohistochemical, and immunofluorescence analyses.

### 2.6. ELISA

Serum was separated, and the concentrations of follicle-stimulating hormone (FSH), estradiol (E2), and anti-Müllerian hormone (AMH) were measured using enzyme-linked immunosorbent assay (ELISA) (Elabscience Biotechnology Inc., Houston, TX, USA).

### 2.7. Gene Expression Analysis

Total RNA was extracted from ovarian tissue samples (Atlas Biotechnology Co., Ltd., Ankara, Türkiye), and complementary DNA (cDNA) synthesis was performed according to the manufacturer’s protocol (Bio-Rad Laboratories, Hercules, CA, USA). Quantitative real-time PCR was conducted using BlasTaq™ 2X qPCR MasterMix with SYBR Green dye (Applied Biological Materials Inc., Richmond, BC, Canada) on a LightCycler 480 PCR system (Roche Diagnostics International Ltd., Rotkreuz, Switzerland). The expression levels of genes associated with ovarian function (*Fshr, Amhr2, and Esr1*), apoptosis (*Casp3*), and oxidative stress and mitochondrial function (*Nfe2l2* (*Nrf2*), *Sod2*, *Slc16a3* (*Mct4*), and *Tomm20*) were evaluated. The primer sequences are provided in Table 1. Relative expression levels of all target genes were normalized to the reference gene Actb. Relative mRNA expression levels were calculated using the 2^−ΔΔCt^ method [5].

### 2.8. Ovarian Reserve Evaluation and Immunocytochemistry

#### 2.8.1. Histological Evaluation

Formalin-fixed ovarian tissues were embedded in paraffin and serially sectioned at a thickness of 5 μm. Every fifth section was systematically collected and stained with hematoxylin and eosin (H&E). To minimize the likelihood of duplicate counting across consecutive sections, only follicles containing a clearly identifiable oocyte nucleus were included in the analysis. Follicles identified in the systematically sampled sections were counted, and the cumulative count was multiplied by the sampling interval (five) to obtain an estimated follicle number per ovary, as previously described [23]. The same sectioning interval, follicle identification criteria, counting procedure, and correction factor were applied consistently to all experimental groups. Accordingly, the reported follicle numbers represent estimates based on systematic histological sampling rather than unbiased stereological measurements of the absolute ovarian follicle population. Based on previously established criteria, follicles were categorized into developmental stages: primordial, primary, secondary, Graafian follicles, corpus luteum, and atretic follicles [5,24].

#### 2.8.2. Light Microscopic Immunohistochemical Analysis

Proliferative and apoptotic activity were evaluated by light microscopy (Leica DM1000), using proliferating cell nuclear antigen (PCNA) as a marker of cellular proliferation and Caspase-3 as a marker of apoptosis. Paraffin-embedded ovarian tissue sections were incubated with primary antibodies against PCNA (Elabscience, Cat. No. E-AB-22001, Houston, TX, USA; 1:200) and Caspase-3 (Invitrogen, Cat. No. 43-7800, clone 74T2, Thermo Fisher Scientific, Waltham, MA, USA; 1:50), followed by the Mouse and Rabbit Specific HRP/DAB IHC Detection Kit (Abcam, Cat. No. ab236466, Cambridge, UK). Mayer’s hematoxylin was used as a counterstain. Immunoreactivity was evaluated under a light microscope, and the findings were compared between the experimental and control groups [25].

#### 2.8.3. Immunofluorescence Staining

Ovarian tissue sections underwent deparaffinization, rehydration, and permeabilization using phosphate-buffered saline (PBS) with Triton X-100. To prevent non-specific binding, an appropriate blocking solution was applied. Subsequently, the sections were incubated overnight at 4 °C with primary antibodies diluted in PBS, specifically anti-Caspase-3 (Abcam, ab92552, 1:500) or anti-Ki-67 (Abcam, ab15580, 1:200), each applied to separate tissue sections. Following extensive washing with PBS, the sections were incubated for 1 h at room temperature with the corresponding fluorescent secondary antibodies: goat anti-rabbit IgG (Texas Red, Abcam, ab6719, 1:1000) for Caspase-3 staining or donkey anti-rabbit IgG (Alexa Fluor 488, Abcam, ab150073, 1:1000) for Ki-67 staining. After the final washing steps, nuclei were counterstained with DAPI using a mounting medium containing the fluorochrome (UltraCruz, TX, USA), and coverslips were applied. Fluorescent images were acquired using a confocal laser scanning microscope (Leica SP8 Lightning, Germany), and signal intensities were analyzed to assess cellular proliferation and apoptosis [23].

### 2.9. Statistical Analysis

No animals met these circumstances during the study; all 35 animals completed the experiment and were included in the final analyses. Statistical analyses were performed using SPSS version 27.0 (IBM Corp., Armonk, NY, USA), with seven animals per group. The distribution of each variable was assessed with the Shapiro–Wilk test. Serum hormone concentrations and relative gene expression values did not meet the assumption of normality and were compared across the five experimental groups using the Kruskal–Wallis test, followed by Dunn’s post hoc test with Bonferroni correction for multiple comparisons. Follicle counts at each developmental stage and quantitative immunofluorescence measurements obtained with ImageJ software (version 1.54i, National Institutes of Health, Bethesda, MD, USA) were normally distributed and satisfied the assumption of homogeneity of variance (Levene’s test, *p* > 0.05); these data were therefore compared across the five experimental groups using one-way ANOVA followed by Tukey’s HSD post hoc test. All data are presented as mean ± standard deviation. A *p* value below 0.05 was considered statistically significant, and significance levels are indicated in the figures as * *p* < 0.05, ** *p* < 0.01, and *** *p* < 0.001; the comparison group corresponding to each symbol is specified in the legend of the respective figure.

## 3. Results

### 3.1. Characterization of Isolated Mitochondria and Mitochondria-Loaded Alginate Capsules

**Figure 1:**
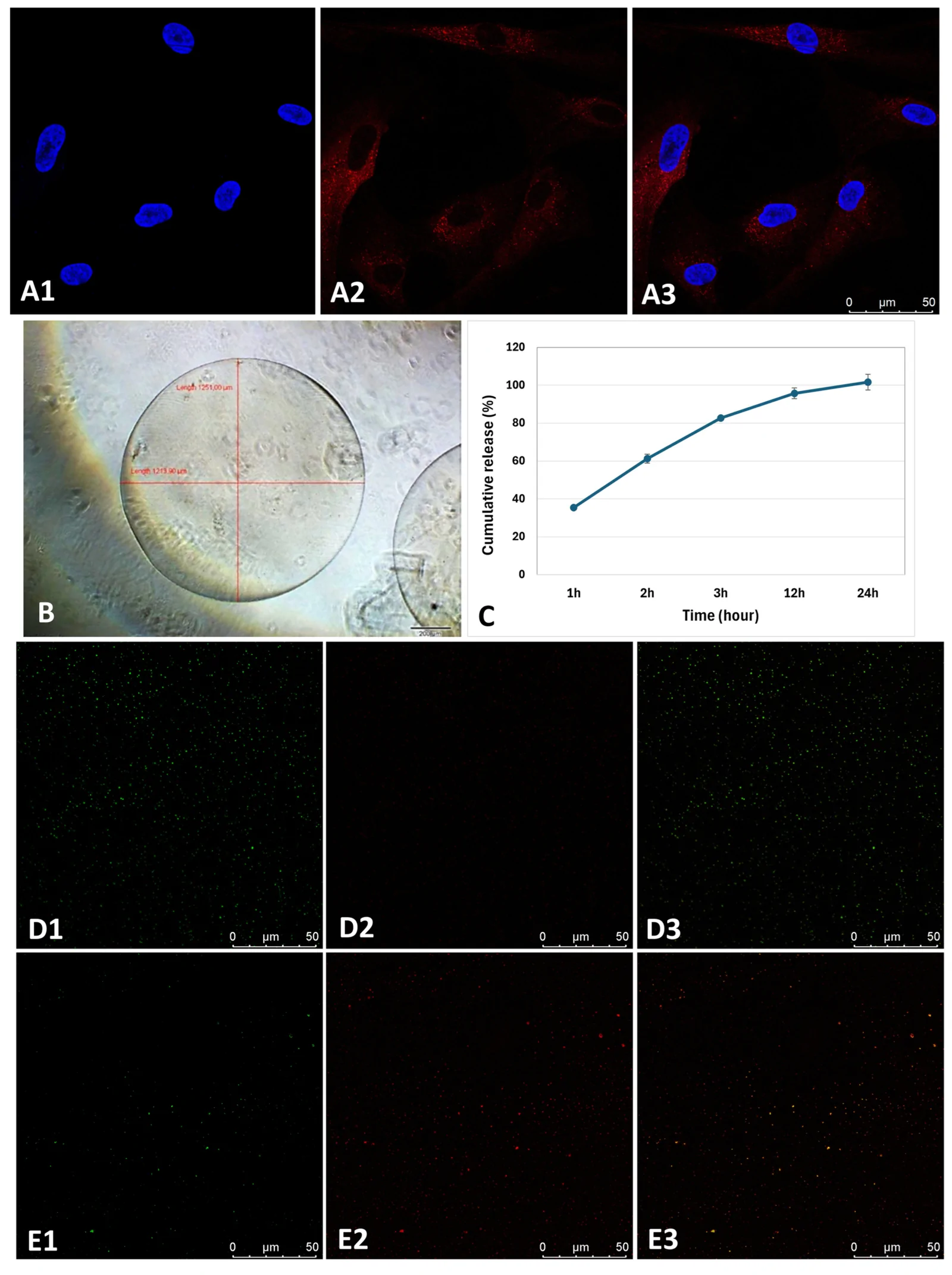
Characterization of isolated mitochondria and mitochondria-loaded alginate capsules. (**A1**) Representative confocal image of intact rBM-MSCs with DAPI-stained nuclei (blue). (**A2**) Representative confocal image of TMRM fluorescence (red) in the same cells, indicating polarized mitochondria prior to isolation. (**A3**) Merged image of DAPI and TMRM fluorescence. (**B**) Representative bright-field image of a mitochondria-loaded alginate capsule. (**C**) In vitro cumulative release profile of mitochondria-associated proteins from alginate capsules over 24 h. Data are presented as mean ± SD (n = 3). (**D1**–**D3**,**E1**–**E3**) JC-1 evaluation of mitochondrial membrane potential in alginate-encapsulated mitochondria. Isolated mitochondria were either heat-treated at 56 °C for 30 min to induce membrane depolarization (**D1**–**D3**) or maintained without heat treatment to preserve mitochondrial membrane potential (**E1**–**E3**). Following JC-1 staining, mitochondria were incorporated into alginate constructs and examined by confocal microscopy. For each condition, green JC-1 monomer fluorescence (**D1**,**E1**), red JC-1 aggregate fluorescence (**D2**,**E2**), and the corresponding merged image (**D3**,**E3**) are shown. Heat-treated mitochondria exhibited predominantly green JC-1 fluorescence with minimal red fluorescence (**D1**–**D3**), consistent with loss of mitochondrial membrane polarization. In contrast, non-heat-treated mitochondria displayed prominent red JC-1 aggregate fluorescence and relatively limited green fluorescence (**E1**–**E3**), indicating preservation of ΔΨm. These findings qualitatively confirmed the presence of membrane-polarized mitochondria within the alginate constructs. Scale bars: 50 µm (**A1**–**A3**,**D1**–**E3**) and 200 µm (**B**).

TMRM staining of intact rBM-MSCs showed a strong perinuclear fluorescence signal, confirming that the donor cells harbored polarized mitochondria before isolation (Figure 1(A1–A3)). The membrane potential of the isolated mitochondria themselves was subsequently assessed by JC-1 staining after encapsulation (see below).

Phase-contrast microscopy showed that the mitochondria-loaded alginate capsules exhibited a uniform spherical morphology with smooth, well-defined surfaces and no visible structural defects such as fragmentation or collapse (Figure 1B). Measurements along two perpendicular axes revealed an average capsule diameter of 1.311 ± 0.127 mm, indicating successful and reproducible capsule fabrication.

The in vitro release assay demonstrated a progressive release of mitochondria-associated proteins from the alginate capsules over 24 h (Figure 1C). Approximately 35% of the encapsulated protein was released within the first hour, followed by cumulative release values of approximately 61%, 83%, and 95% at 2, 3, and 12 h, respectively. Protein release reached completion (≈100%) by 24 h, indicating that the alginate capsules provided a gradual, extended release rather than an immediate burst release of mitochondria-associated proteins.

JC-1 staining demonstrated that the isolated mitochondria retained their membrane potential following incorporation into the alginate constructs. Heat-treated mitochondria exhibited predominantly green JC-1 fluorescence with minimal red fluorescence, consistent with loss of mitochondrial membrane polarization (Figure 1(D1–D3)). In contrast, non-heat-treated mitochondria displayed prominent red JC-1 aggregate fluorescence and relatively limited green fluorescence, indicating preservation of ΔΨm (Figure 1(E1–E3)). These findings qualitatively confirmed the presence of membrane-polarized mitochondria within the alginate constructs.

### 3.2. Effects of Treatment on Serum Hormone Levels

**Figure 2:**
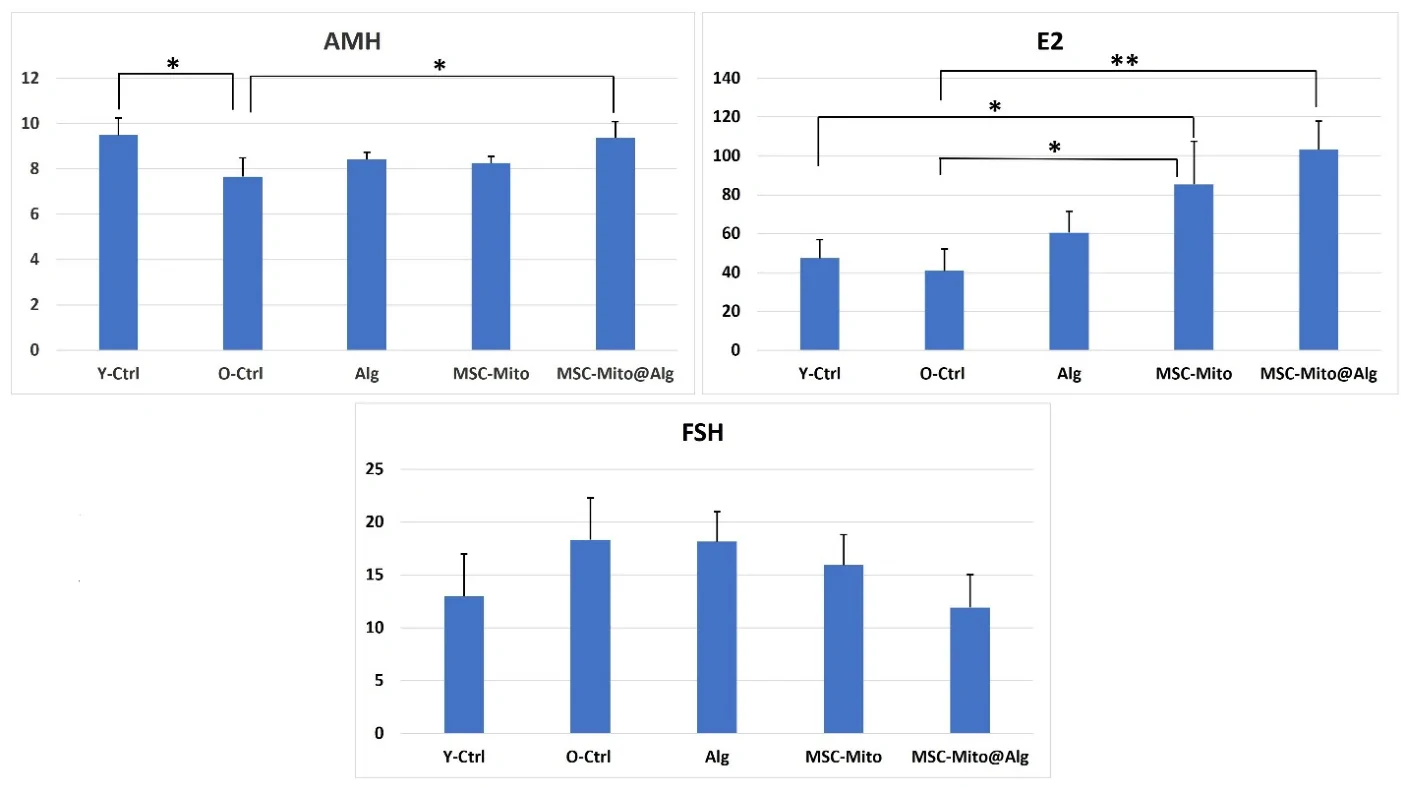
Bar graphs showing changes in AMH, E2, and FSH protein levels measured by ELISA across the experimental groups. AMH and E2 concentrations are expressed as ng/mL, while FSH levels are expressed as mIU/mL. Data are presented as mean ± SD. * *p* < 0.05, ** *p* < 0.01. Significance brackets indicate statistically significant pairwise comparisons. AMH levels were significantly lower in the old control group compared with the young control group (* *p* < 0.023) and were significantly higher in the MSC-Mito@Alg group (* *p* < 0.035) compared with the old control group and no longer differed significantly from the young control group. E2 was significantly higher in the MSC-Mito@Alg group compared with the old control group (*p* = 0.008), with E2 levels also significantly higher, though to a lesser extent, in the MSC-Mito group compared with the old control group (* *p* < 0.046). Although lower FSH levels were observed in the MSC-Mito@Alg and young control groups compared with the old control group, these differences did not reach statistical significance.

Serum hormone analysis revealed significant differences in AMH, E2, and FSH levels among the experimental groups (Figure 2).

### 3.3. Quantitative Real-Time PCR Analysis

**Figure 3:**
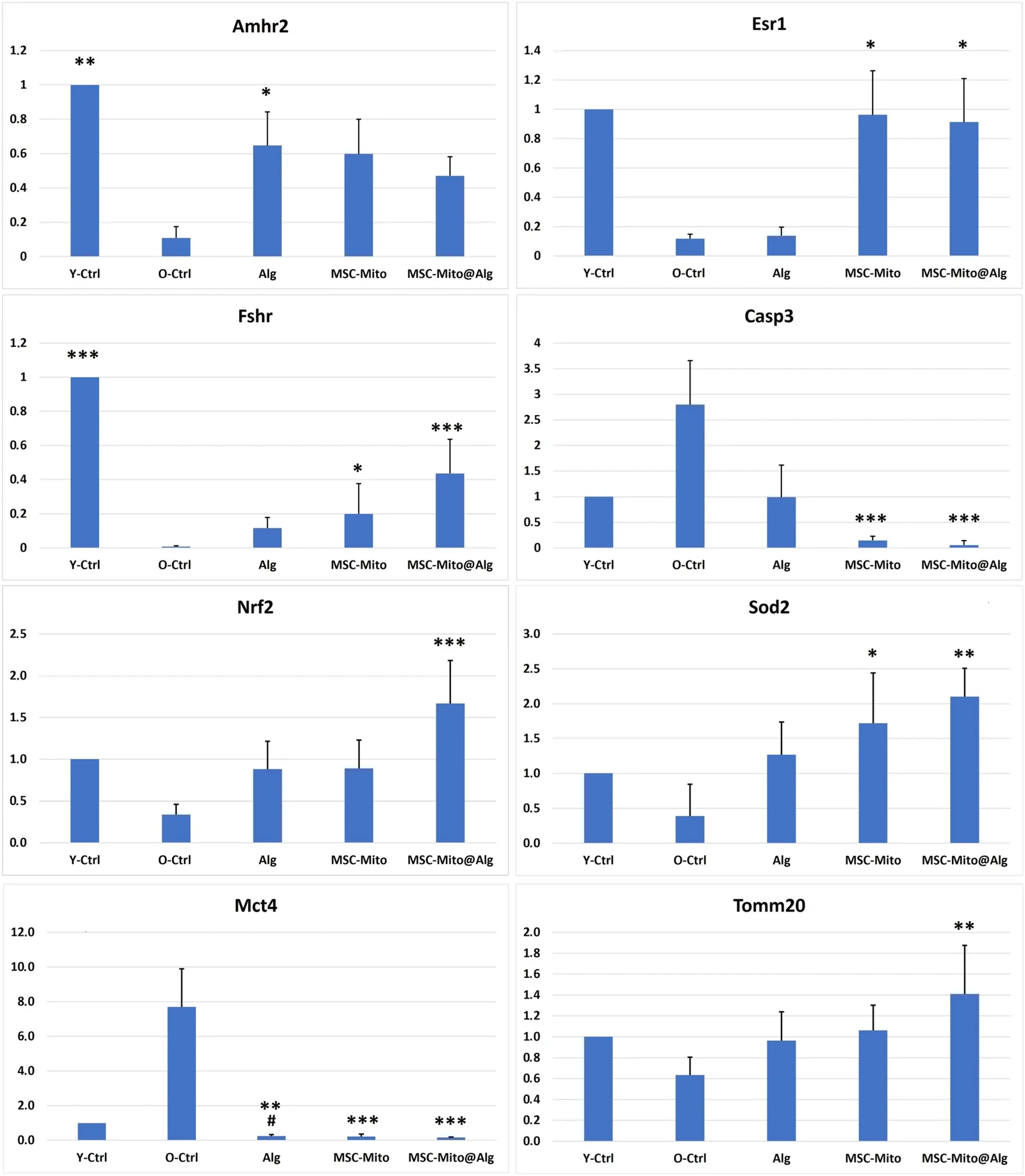
Relative gene expression analysis in ovarian tissues was determined by qRT-PCR. Relative expression levels of Amhr2, Esr1, Fshr, Casp3, Nrf2, Sod2, Mct4, and Tomm20, normalized to Actb. Data are presented as mean ± SD. * *p* < 0.05, ** *p* < 0.01, and *** *p* < 0.001 versus O-Ctrl; # *p* < 0.05 versus Y-Ctrl. Only statistically significant pairwise comparisons are indicated.

Gene expression analysis revealed significant age-related alterations in ovarian tissues (Figure 3). Compared with the young control group, the old control group exhibited significantly lower expression of *Amhr2* and *Fshr* (*p* = 0.005 and *p* < 0.001, respectively). Relative to the old control, *Amhr2* expression was significantly higher in the alginate group (* *p* < 0.028). Notably, *Esr1* and *Fshr* expression levels were both significantly higher in the MSC-Mito group (*p* = 0.046 for both), with the effect on *Fshr* most pronounced in the MSC-Mito@Alg group (*p* < 0.001). Conversely, *Casp3* expression was significantly lower in both the MSC-Mito and MSC-Mito@Alg groups compared with the old control (*** *p* < 0.001), indicating reduced age-associated apoptotic signaling (Figure 3).

Gene expression analysis demonstrated that mitochondrial treatment significantly modulated antioxidant- and metabolic stress-related transcripts. *Nrf2* and *Tomm20* expression were each significantly higher only in the MSC-Mito@Alg group relative to the old control (*p* < 0.001 and *p* = 0.003, respectively). *Sod2* expression was significantly higher in both the MSC-Mito (*p* = 0.021) and MSC-Mito@Alg (*p* < 0.001) groups relative to the old control. *Mct4* expression was significantly lower in the Alg (*p* = 0.008), MSC-Mito (*p* < 0.001), and MSC-Mito@Alg (*p* < 0.001) groups relative to the old control; MSC-Mito@Alg also differed significantly from the young control group (*p* = 0.014). Overall, mitochondrial treatment was associated with higher hormone receptor and antioxidant transcripts, lower *Casp3* and *Mct4*, and higher *Tomm20* expression relative to old control, with *Nrf2* and *Tomm20* responding only in the MSC-Mito@Alg group.

### 3.4. Histology and Immunohistochemistry

**Figure 4:**
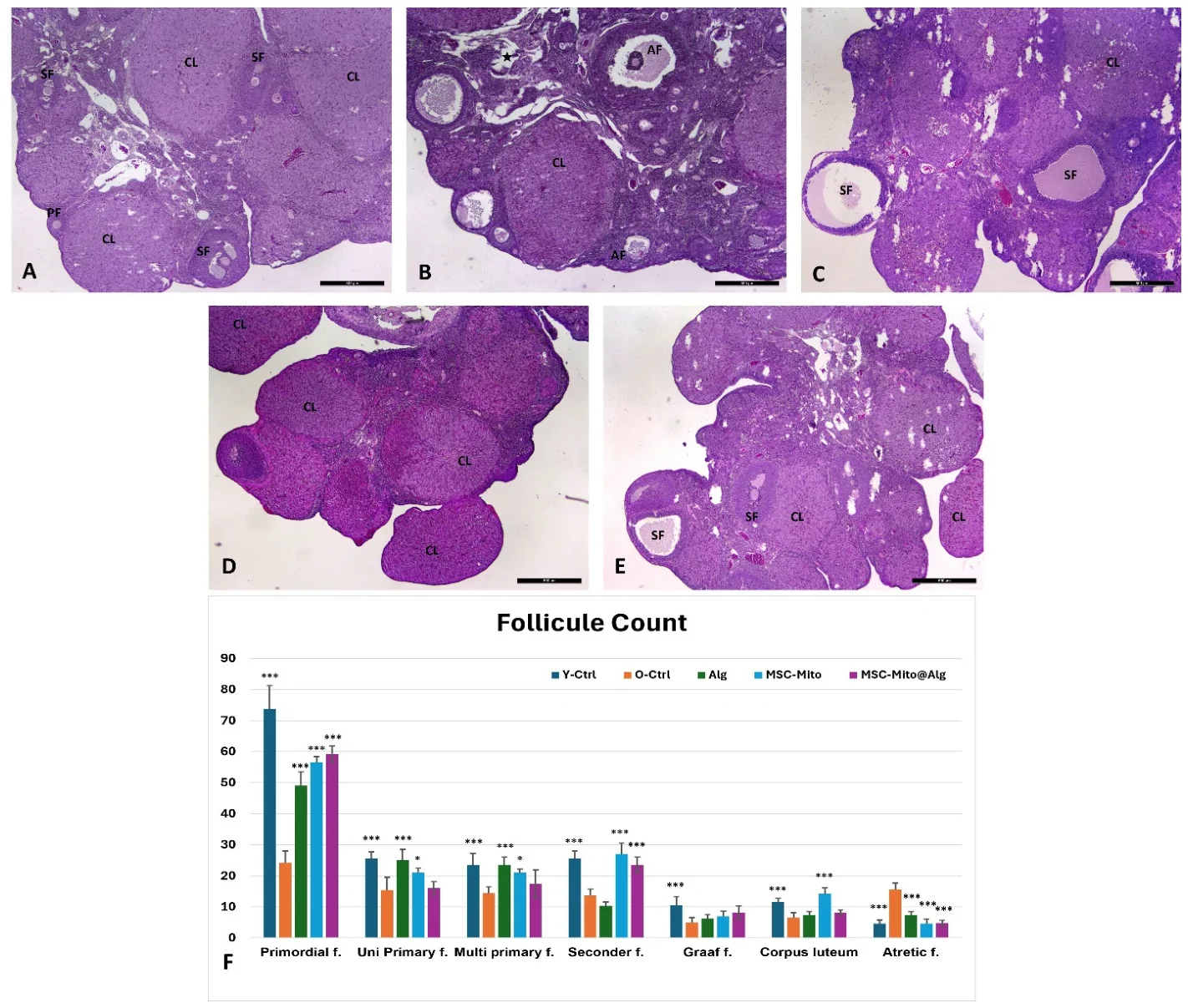
Representative H&E-stained ovarian sections from the young control (**A**), old control (**B**), alginate control (**C**), MSC-Mito (**D**), and MSC-Mito@Alg (**E**) groups. PF: primordial/primary follicle; SF: secondary follicle; AF: antral follicle; CL: corpus luteum; ★: interstitial edema. Scale bars = 500 µm. (**F**) Follicle counts at each developmental stage across the five experimental groups. Data are mean ± SD; one-way ANOVA with Tukey’s HSD post hoc test vs. O-Ctrl. * *p* < 0.05, *** *p* < 0.001. Scale bars = 500 µm.

Histological evaluation of ovarian sections by H&E staining revealed marked differences in follicular architecture among the experimental groups (Figure 4). The young control group (Figure 4A) exhibited well-preserved ovarian morphology, with follicles at different developmental stages, including primordial/primary follicles (PF), secondary follicles (SF), and abundant corpus luteum (CL), indicating active folliculogenesis and a well-preserved follicular reserve. In contrast, the old control group (Figure 4B) showed disrupted ovarian architecture, with fewer developing follicles, degenerative changes, and interstitial edema, consistent with age-related ovarian decline. Although antral follicles (AF) and CL structures were still observed, overall follicular organization was lower compared with the young group. The alginate control group (Figure 4C) demonstrated only limited improvement, with sparse follicular development and less organized ovarian tissue. In the MSC-Mito-treated group (Figure 4D), follicular organization was improved, with better-preserved ovarian morphology and higher follicular development. Notably, the MSC-Mito@Alg group (Figure 4E) showed the most prominent histological recovery, characterized by enhanced folliculogenesis, the presence of developing follicles, and a better-preserved ovarian architecture compared with the old and alginate control groups.

Follicle numbers at each developmental stage were quantified across the five experimental groups (Figure 4F). One-way ANOVA revealed significant differences among groups for all follicle classes (*p* ≤ 0.001), with Tukey’s HSD used to compare each group against the aged control (O-Ctrl).

O-Ctrl animals showed a markedly lower number of primordial follicles compared with Y-Ctrl (24.2 ± 3.8 vs. 73.8 ± 7.5; *p* < 0.001) and a correspondingly higher number of atretic follicles (15.6 ± 2.1 vs. 4.6 ± 1.1; *p* < 0.001), confirming the aging model. Primordial follicle numbers were significantly higher than O-Ctrl in all treatment groups (Alg, MSC-Mito, MSC-Mito@Alg; *p* < 0.001 for all), with the highest counts in the mitochondria-treated groups.

Secondary follicle numbers were significantly higher in both the MSC-Mito (27.0 ± 3.5) and MSC-Mito@Alg (23.6 ± 2.4) groups than in O-Ctrl (13.6 ± 2.1; *p* < 0.001 for both), whereas Alg alone did not differ (*p* = 0.237), consistent with an effect attributable to the mitochondrial treatment rather than the carrier alone. At the primary follicle stage, MSC-Mito@Alg did not differ from O-Ctrl (*p* > 0.49), whereas Alg and MSC-Mito did (*p* ≤ 0.037). Graafian follicle numbers did not differ significantly between O-Ctrl and any treatment group, whereas corpus luteum counts were significantly higher only with MSC-Mito (14.2 ± 1.9 vs. 6.6 ± 1.5; *p* < 0.001). Atretic follicle counts were significantly lower relative to O-Ctrl in all treatment groups (*p* < 0.001), reaching levels comparable to Y-Ctrl.

**Figure 5:**
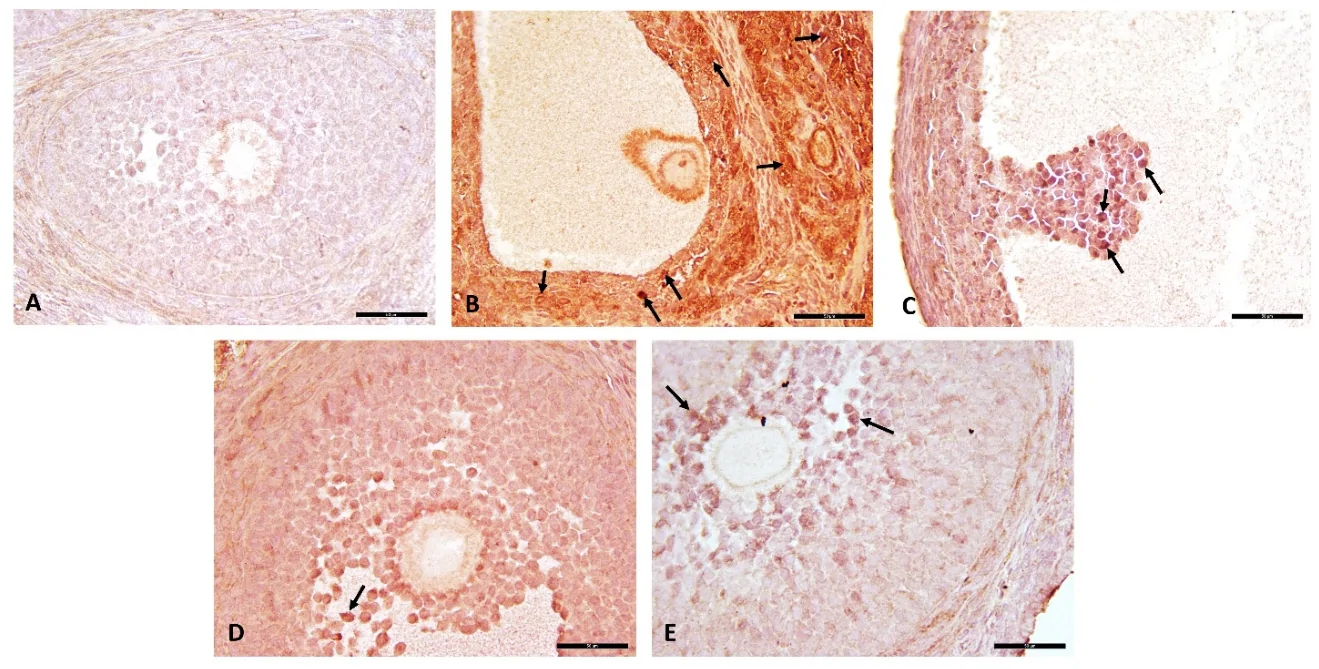
Caspase-3 immunohistochemical staining in the young control (**A**), old control (**B**), alginate control (**C**), MSC-derived mitochondria alone (MSC-Mito) (**D**), and alginate-encapsulated MSC-derived mitochondria (MSC-Mito@Alg) (**E**). Arrows indicate Caspase-3-positive cells. Scale bars = 50 µm.

Immunohistochemical analysis of Caspase-3 was performed to assess apoptotic activity in ovarian tissues across the experimental groups (Figure 5). In the young control group (Figure 5A), Caspase-3 positive cells were rarely observed, indicating a low basal level of apoptosis in healthy ovarian follicles. In contrast, the old control group (Figure 5B) exhibited higher Caspase-3 expression, particularly within the granulosa cell layers (black arrows), suggesting enhanced apoptotic activity associated with ovarian aging.

In the alginate control group (Figure 5C), Caspase-3 immunoreactivity was observed in clusters of granulosa cells adjacent to the follicular lumen. Treatment with MSC-derived mitochondria (MSC-Mito) (Figure 5D) resulted in lower Caspase-3 positive staining, particularly around early antral follicles (black arrows). The MSC-Mito@Alg group (Figure 5E) demonstrated the lowest Caspase-3 immunoreactivity, with only a few isolated positive cells detected within the follicular wall and antral space.

**Figure 6:**
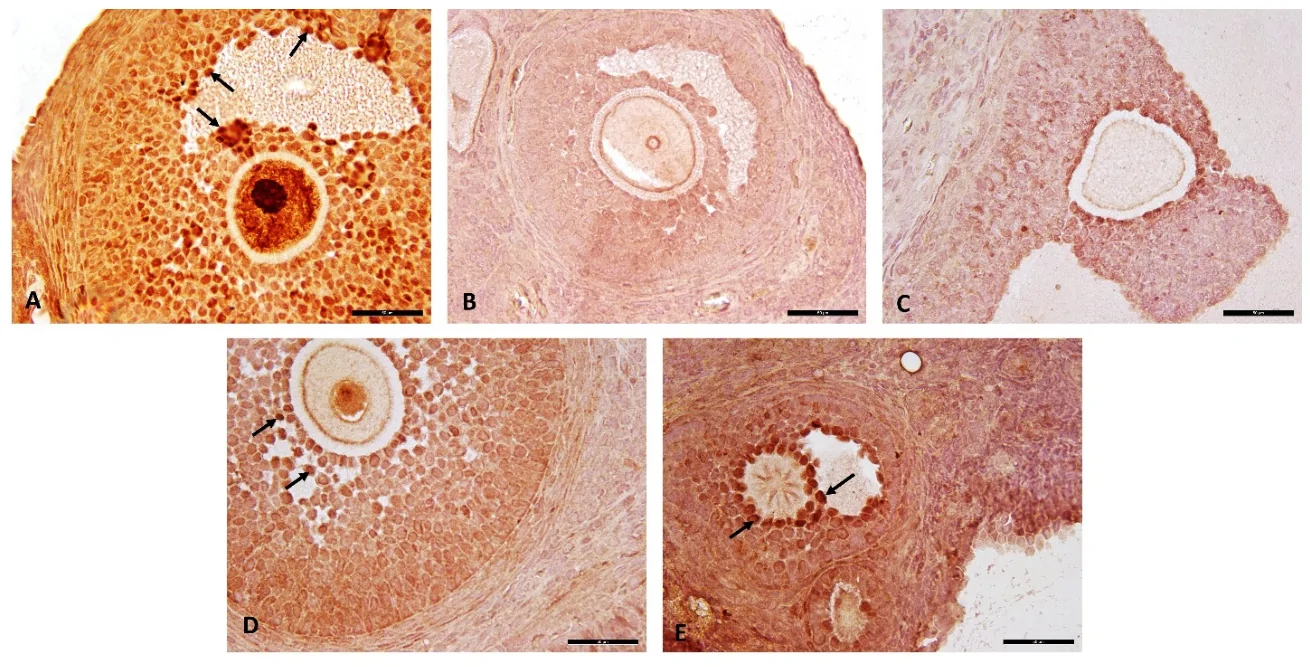
PCNA IHC staining in the young control (**A**), old control (**B**), alginate control (**C**), MSC-Mito (**D**), and MSC-Mito@Alg (**E**). Arrows indicate PCNA-positive cells. Scale bars = 50 µm.

Immunohistochemical staining for PCNA was conducted to assess proliferative activity in ovarian tissues across the experimental groups (Figure 6). In the young control group (Figure 6A), strong nuclear PCNA expression was observed in granulosa and theca cells (arrows), indicating active folliculogenesis within the follicles. Conversely, the old control group (Figure 6B) displayed markedly lower PCNA positivity, suggesting diminished proliferative activity with aging. The alginate control group (Figure 6C) exhibited weak PCNA staining localized mainly to perifollicular regions. However, treatment with MSC-Mito (Figure 6D) was associated with a higher number of PCNA-positive cells, particularly within the granulosa cell compartments of developing follicles (black arrows). The MSC-Mito@Alg group (Figure 6E) demonstrated the most pronounced PCNA immunoreactivity, with strong staining observed in granulosa cells surrounding developing follicles.

**Figure 7:**
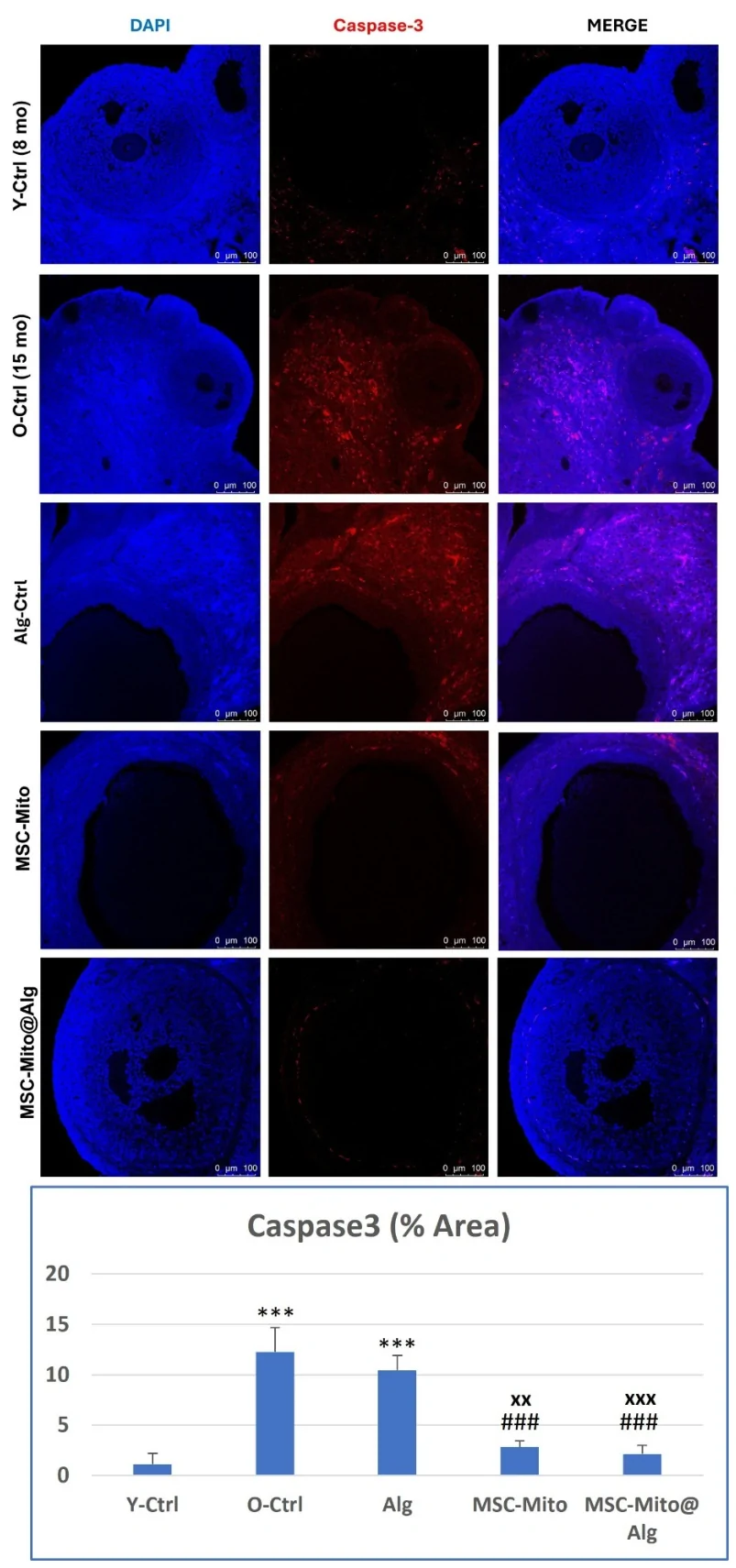
Representative immunofluorescence images and quantitative analysis of Caspase-3 expression in ovarian tissues from the young control (Y-Ctrl, 8 mo), old control (O-Ctrl, 15 mo), alginate control (Alg-Ctrl), MSC-Mito, and MSC-Mito@Alg groups. Nuclei were counterstained with DAPI (blue), Caspase-3-positive areas are shown in red, and merged images are presented in the right panel. Quantitative analysis of Caspase-3-positive area (%) was performed using ImageJ. Data are presented as mean ± SD. *** *p* < 0.001 vs. Y-Ctrl; xx *p* < 0.01, xxx *p* < 0.001 vs. O-Ctrl; ### *p* < 0.001 vs. Alg-Ctrl. Scale bars = 100 µm.

**Figure 8:**
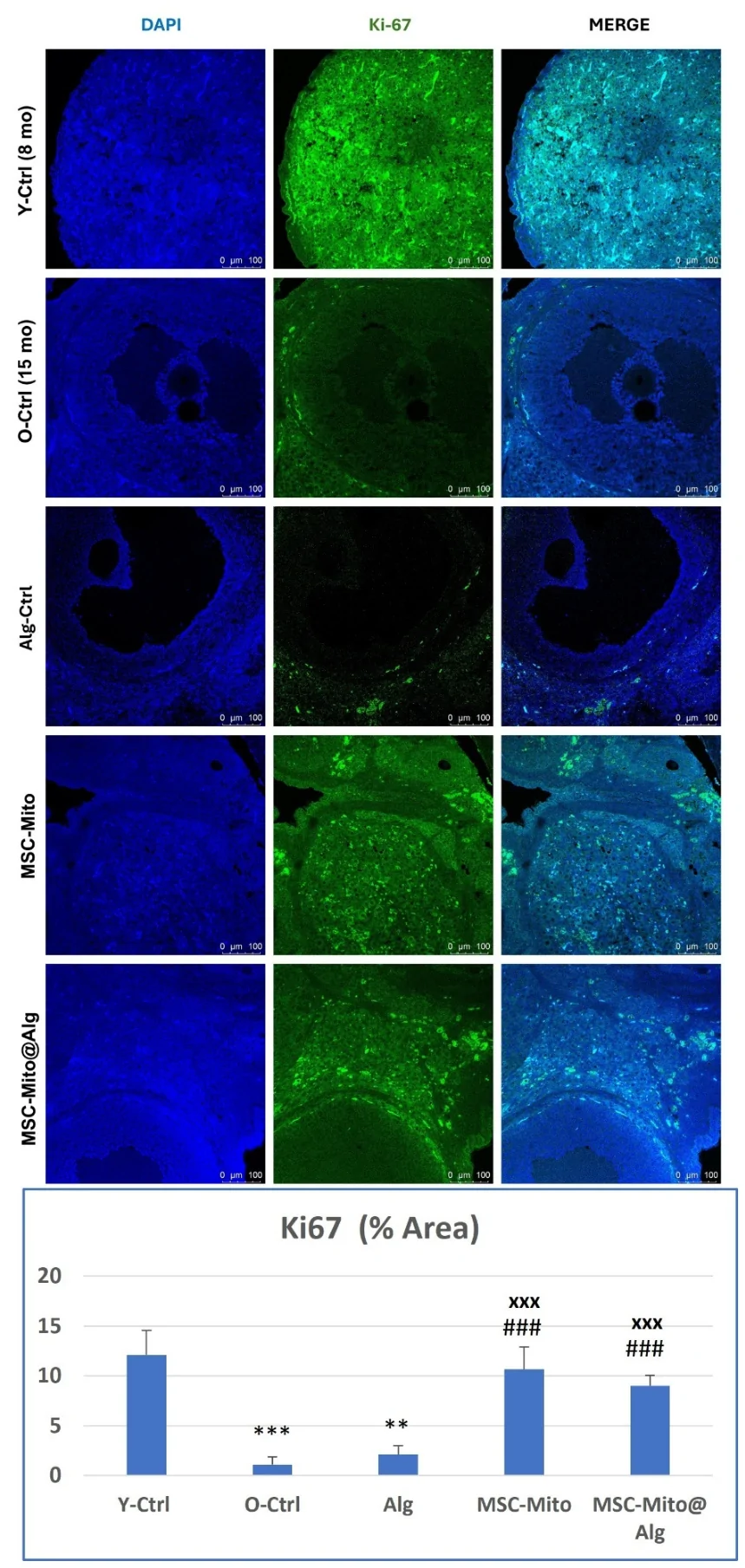
Representative immunofluorescence images and quantitative analysis of Ki-67 expression in ovarian tissues from the young control (Y-Ctrl, 8 mo), old control (O-Ctrl, 15 mo), alginate control (Alg-Ctrl), MSC-Mito, and MSC-Mito@Alg groups. Nuclei were counterstained with DAPI (blue), Ki-67-positive areas are shown in green, and merged images are presented in the right panel. Quantitative analysis of the Ki-67-positive area (%) was performed using ImageJ. Data are presented as mean ± SD. ** *p* < 0.01, *** *p* < 0.001 vs. Y-Ctrl; xxx *p* < 0.001 vs. O-Ctrl; ### *p* < 0.001 vs. Alg-Ctrl. Scale bars = 100 µm.

Immunofluorescence analysis revealed reciprocal alterations in apoptotic and proliferative markers among the experimental groups. The caspase-3-positive area was higher in the old control (12.27 ± 2.41%) and alginate control (10.45 ± 1.47%) groups compared with the young control group (1.12 ± 1.09%) (Figure 7), whereas the Ki-67-positive area was lower in these groups (1.08 ± 0.76% and 2.11 ± 0.90%, respectively, vs. 12.08 ± 2.48% in the young control; * *p* < 0.05) (Figure 8). In contrast, treatment with MSC-Mito (Caspase-3: 2.82 ± 0.62%; Ki-67: 10.65 ± 2.26%) and MSC-Mito@Alg (Caspase-3: 2.16 ± 0.84%; Ki-67: 9.01 ± 1.04%) was associated with lower Caspase-3-positive areas and higher Ki-67-positive areas compared with the old control (xp < 0.05) and alginate.

Quantitative analyses of immunofluorescence images performed using ImageJ software confirmed that mitochondrial-based treatments were associated with lower apoptotic signaling and higher proliferative activity in the aged ovarian tissue.

## 4. Discussion

Mitochondrial dysfunction is a hallmark of physiological aging and is closely associated with the development of various age-related degenerative conditions. Declines in mitochondrial quality and activity contribute to aging by promoting cellular senescence, chronic inflammation, and impaired stem cell function [13]. These alterations are particularly relevant in the ovary, where aging is characterized by disrupted folliculogenesis, hormonal imbalance, and increased apoptotic activity. Mitochondria play a critical role in ovarian physiology by regulating cellular energy metabolism, steroidogenesis, and oocyte competence; therefore, age-related mitochondrial dysfunction has been proposed as a key driver of declining ovarian reserve and impaired follicular development.

Targeted delivery of therapeutic agents to the ovary or its immediate microenvironment has emerged as a promising strategy to enhance treatment efficacy while minimizing systemic side effects. Previous studies in rodent models have demonstrated that the direct administration of regenerative agents to the ovarian region can enhance ovarian function [20]. Furthermore, anatomical and functional studies have identified the peri-ovarian region as a distinct microenvironment contributing to folliculogenesis and ovarian homeostasis. Peri-ovarian adipose tissue and surrounding stromal structures have been shown to influence follicular development and endocrine signaling in rodent models [21]. In accordance with this concept, both free isolated mitochondria and alginate-encapsulated mitochondrial formulations in the present study were administered peri-ovarianly to ensure comparable local exposure of ovarian tissue. Surgical placement was required only for the alginate-based formulation because the solid structure of the carrier system cannot be delivered through a needle. The free mitochondrial suspension was instead injected percutaneously so that the peri-ovarian compartment was not opened and dispersion of the liquid away from the ovarian surface was limited. The two routes therefore follow from the physical properties of each formulation. Hydrogel-based delivery systems placed around ovarian tissue have been reported to modify local delivery kinetics and prolong tissue exposure, thereby improving therapeutic retention compared with liquid formulations [9].

The functional integrity of the isolated mitochondria was confirmed by JC-1 staining after encapsulation, which showed preservation of mitochondrial membrane potential. This assay demonstrates that mitochondrial viability was preserved at the time of encapsulation; it does not demonstrate uptake of the administered mitochondria by ovarian cells or functional integration in vivo, which was not directly assessed in this study (see Section 5). The release kinetics, however, require careful interpretation. Approximately 35% of the encapsulated protein was released within the first hour, and cumulative release increased progressively thereafter, reaching completion only by 24 h. This more gradual profile indicates that the alginate capsules functioned as a short-to-intermediate-term reservoir, providing extended local exposure over the first day rather than an immediate burst release, but still falling far short of the 14-day interval over which therapeutic outcomes were assessed. Consequently, the therapeutic advantage of MSC-Mito@Alg observed on day 14 cannot be attributed to continuous release throughout the entire experimental period. A more plausible interpretation is that encapsulation shaped the early-to-intermediate phase of exposure: concentrating the mitochondrial preparation into a solid matrix placed directly in the peri-ovarian space is likely to have limited immediate dispersion into the surrounding tissue and peritoneal fluid, yielding higher and more homogeneous local exposure during the first 24 h after administration. Since the capsules were positioned in direct contact with the ovarian surface, this extended early exposure window may have been sufficient to initiate the downstream tissue responses of restored hormone receptor signaling, higher antioxidant capacity, and lower apoptotic activity that were still detectable two weeks later. Direct evidence for this proposed mechanism was not obtained in the present study and remains to be tested using labeled mitochondria and later sampling time points.

Zhang and colleagues investigated the effect of alginate-encapsulated menstrual blood-derived mesenchymal stromal cells (MenSCs) and their mitochondria on ovarian function in aged mice and reported beneficial effects in both applications [9]. Their findings demonstrated enhanced folliculogenesis, increased AMH, FSH, and E2 levels, upregulation of ovarian function-related genes (*Fshr*, *Amhr2*, and *Esr1*), and downregulation of the apoptosis-related gene *Casp3*, supporting the therapeutic potential of mitochondrial delivery within alginate matrices.

Building on that work, the present study addresses a question that has not previously been tested directly: whether hydrogel encapsulation itself alters the therapeutic performance of exogenous mitochondria in the aged ovary. Rather than comparing mitochondrial treatment with untreated aging alone, we examined free and alginate-encapsulated mitochondria head-to-head within the same experiment, using a bone marrow-derived rat MSC source, an empty hydrogel control group, and a physiological rat aging model. Moreover, MSC-Mito@Alg treatment was associated with higher *Nrf2* expression, while both mitochondrial treatment groups showed higher *Sod2* expression, and *Tomm20* expression was higher only in the MSC-Mito@Alg group.

Reciprocal regulation of Caspase-3 and Ki-67 further indicates that mitochondrial supplementation may be associated with lower apoptotic signaling and higher cellular proliferative capacity, thereby contributing to restoration of ovarian tissue homeostasis. Encapsulating mesenchymal stromal cells within hydrogel systems such as alginate has been shown to provide three-dimensional structural support, enhance metabolic activity, and protect cells from immune-mediated damage. Additionally, alginate-based encapsulation enables controlled release and regulation of biologically active factors secreted by MSCs [14]. In a rat model of liver cirrhosis, delivery of MSCs within an alginate hydrogel promoted liver regeneration while significantly reducing fibrosis. Furthermore, hydrogel-based MSC administration improved cell retention and therapeutic efficacy, thereby supporting tissue repair and functional recovery [26].

Recent studies have suggested that encapsulation of MSC-derived mitochondria within alginate/gel matrices can improve mitochondrial stability, facilitate controlled release, and enhance targeted delivery in mitochondrial-based therapeutic strategies [17]. Alginate matrices can reduce cargo loss while enabling localized and sustained delivery of therapeutic components. The advantages of alginate-based microencapsulation, including improved retention, immune protection, and sustained biological activity, have been widely described [15]. Consistently, encapsulation within alginate hydrogels has been reported to improve post-transplantation survival and therapeutic efficacy [16]. The differences observed following MSC-Mito@Alg treatment may, at least in part, reflect the more gradual release of mitochondria-associated components over the first 24 h observed in our in vitro assay and the expected greater local retention of the solid capsule matrix compared with a free suspension. However, because the two formulations required different administration procedures, procedure-related factors, including local tissue manipulation, inflammatory responses, and differences in local exposure, may also have contributed to these effects. Therefore, the present study cannot distinguish the contribution of alginate encapsulation itself from that of the associated administration procedure. Encapsulation was not uniformly associated with greater effects, however: corpus luteum counts, secondary follicle numbers, and Ki-67 immunoreactivity were at least as high after free mitochondria, whereas differences favoring MSC-Mito@Alg were mainly observed in antioxidant and mitochondrial-mass endpoints.

One further observation requires comment. The empty-capsule group was not biologically inert: *Amhr2* expression was significantly higher in this group, and *Mct4* expression was lower than the young-control level, indicating that part of the transcriptional response was attributable to the carrier rather than to the delivered mitochondria. Two explanations are plausible. First, alginate is slowly depolymerized in vivo into alginate oligosaccharides, which are not inert degradation products but bioactive molecules with documented ovarian effects. In busulfan-induced premature ovarian insufficiency, alginate oligosaccharide administration restored reproductive hormone concentrations and follicular development [27], and in a D-galactose model it normalized serum hormones, reduced granulosa cell apoptosis, increased follicle counts, and lowered ovarian oxidative stress [28]; a comparable antioxidant action has been linked to Nrf2 signaling in other tissues [29]. These studies, however, used oral administration and attributed much of the effect to the gut microbiota, so an equivalent local action of oligosaccharides released from an implanted capsule remains a hypothesis rather than an established mechanism. Second, *Mct4* is a canonical hypoxia-responsive gene whose transcription is driven by HIF-1α through hypoxia-response elements in its promoter [30], and the lactate–HIF-1α axis is active in granulosa cells [31]; any change in local oxygenation around the implant, such as the neovascularization that accompanies a foreign-body response, would therefore be expected to alter *Mct4* expression independently of the administered mitochondria. It should also be acknowledged that the isolated rise in *Amhr2* in the alginate group was not reproduced in any other transcript and may equally reflect biological variability within a group of seven animals. For these reasons, the present interpretation rests on the consistency of the overall pattern rather than on any single gene: *Esr1*, *Fshr*, *Casp3*, *Nrf2*, and *Tomm20* expressions, hormone concentrations, and immunofluorescence measurements all shifted in the same direction, and the effects that reached significance in a single group were confined to MSC-Mito@Alg, with the exception of corpus luteum counts, which were higher only with free mitochondria.

## 5. Limitations

Several limitations should be considered when interpreting these findings. Follicle numbers were estimated using systematic sampling of every fifth ovarian section rather than an unbiased stereological reconstruction of the entire ovary. Although the same sectioning interval, follicle identification criteria, counting procedure, and correction factor were applied consistently across all experimental groups, this approach provides comparative estimates rather than absolute measurements of the ovarian follicle population. Therefore, the observed differences, particularly the apparent increases in primordial and secondary follicle numbers, should be interpreted as relative differences in estimated follicle abundance between groups rather than as precise changes in the absolute ovarian follicle reserve. The in vitro release assay was performed on constructs prepared at a 1:1 (*v*/*v*) alginate-to-mitochondria ratio rather than on the concentrated formulation implanted in vivo, so the measured kinetics approximate rather than reproduce the release profile of the capsules administered. Similarly, the chromogenic immunohistochemical findings for Caspase-3 (Figure 5) and PCNA (Figure 6) are descriptive rather than quantified, so the magnitude of these specific staining patterns cannot be stated numerically; Caspase-3 and Ki-67 immunoreactivity were, however, separately quantified by immunofluorescence and image analysis (Figure 7 and Figure 8). The in vitro release assay quantified total mitochondria-associated protein rather than intact organelles, and release was essentially complete within 24 h; the kinetics of functionally competent mitochondria and their relationship to the outcomes measured on day 14 therefore remain undetermined. Uptake of the administered mitochondria by ovarian cells was not demonstrated in vivo using labeled organelles. The inclusion of an empty-capsule group excludes a carrier-mediated effect, but the contribution of mitochondria-derived soluble components acting through paracrine mechanisms cannot be formally separated from that of the intact organelles, and this distinction will require labeled-organelle tracking or a heat-inactivated mitochondrial control in future work. The two mitochondrial formulations were delivered by different routes. Because the free mitochondrial preparation was a liquid suspension, administration through an open surgical field could have increased leakage or dispersion away from the target site; it was therefore injected percutaneously without opening the peri-ovarian compartment. In contrast, the solid alginate capsules required surgical placement through a dorsal incision with retroperitoneal access. These procedural differences may themselves influence local tissue injury and inflammatory responses, while the different delivery formats may also affect mitochondrial retention, dispersion, and local exposure. Although the empty-carrier group controlled for effects attributable to the alginate material, it did not control for differences associated with the administration procedure. Therefore, differences observed between the MSC-Mito and MSC-Mito@Alg groups cannot be attributed exclusively to alginate encapsulation, and the administration route represents a potential confounding factor in the direct comparison of these treatments. The free mitochondrial suspension was injected without image guidance, so its exact distribution within the peri-ovarian region could not be confirmed, and the untreated control groups did not undergo sham procedures. In addition, the alginate-only group showed significant changes in some transcripts, indicating that the carrier is not biologically inert and that effects should not be attributed exclusively to the encapsulated mitochondria. Ovarian aging was modeled in 15-month-old animals, and estrous cyclicity was recorded only to standardize the phase at intervention rather than as an outcome measure. Finally, outcomes were evaluated at a single time point, 14 days after administration, and no functional reproductive endpoint such as mating outcome or oocyte yield and quality was assessed; the durability and reproductive relevance of the observed changes therefore remain to be established. Accordingly, the follicular, endocrine, and molecular parameters assessed here should be interpreted as surrogate markers of ovarian aging rather than direct proof of restored reproductive function.

Beyond the limitations noted above, several refinements would strengthen the translational relevance of this experimental model. First, tracking fluorescently labeled mitochondria at defined time points after administration, together with donor-specific mitochondrial DNA quantification and assessment of OXPHOS activity/ATP production in ovarian tissue, would allow direct confirmation of mitochondrial persistence, integration, and functional contribution, addressing the gap left by the absence of direct transfer evidence in the present study. Second, outcomes were assessed at a single 14-day time point; longer-term follow-up is needed to determine whether the observed improvements in surrogate markers are sustained and translate into an extended reproductive window. Finally, systematic dose–response studies comparing different mitochondrial doses and administration schedules would help define the minimum effective and safe dose range before progressing toward further studies.

## 6. Conclusions

The present findings indicate that localized delivery of alginate-encapsulated MSC-derived mitochondria may provide an effective cell-free approach for improving surrogate markers of ovarian function during aging. Within the same experiment, the encapsulated formulation was associated with greater effects on some antioxidant and mitochondrial-mass endpoints but did not outperform free mitochondria across follicular and proliferative outcomes. Although encouraging therapeutic effects were observed, additional preclinical studies are warranted to establish long-term efficacy and further elucidate the underlying mechanisms.

## Figures and Tables

**Table 1:** Forward and reverse primer sequences used for qRT-PCR analysis of target and reference genes.

Gene	Primer Sequences (Forward/Reverse)	Accession Number
Amhr2 (anti-Müllerian hormone receptor type 2)	F- TTCCAAGGAAGCGTTGACGA R- TCCTGTTTGGGGATACTTGTGC	NM_030998.2
Esr1 (estrogen receptor alpha)	F- TTTCTTTAAGAGAAGCATTCAAGGA R- TTATCGATGGTGCATTGGTTT	NM_012689.2
Fshr (follicle-stimulating hormone receptor)	F- CATTCTTGGGCACGGGATCT R- GGTGAGCACAAACCTCAGTTC	NM_199237.2
Casp3 (caspase 3)	F- GTGGAACTGACGATGATATGGC R- CGCAAAGTGACTGGATGAACC	NM_012922.2
*Nfe2l2* (nuclear factor erythroid 2-related factor 2; *Nrf2*)	F- CCCAGCACATCCAGACAGAC R- TAGCTCCTGCCAAACTTGCT	NM_031789.3
Sod2 (superoxide dismutase 2)	F- ACGCGACCTACGTGAACAAT R- CAGGCTGAAGAGCAACCTGA	NM_017051.2
*Slc16a3* (monocarboxylate transporter 4; *Mct4*)	F- CAAAGGGGCACTGATGACAC R- GCGGTTCACACACATACTGC	NM_030834.2
Tomm20 (translocase of outer mitochondrial membrane 20)	F- CCACCAGTGTTCCAGATGCT R- GGTGTCTGGCTCATTCCACA	NM_152935.2
Actb (beta actin)	F- TACAACCTTCTTGCAGCTCC R- CCTTCTGACCCATACCCACC	NM_031144.3

## Data Availability

The datasets generated and analyzed during the current study have not been deposited in a public repository but are retained by the research team and are available from the corresponding author upon request.

## Supplementary Materials

The following supporting information can be downloaded at: https://www.mdpi.com/article/10.3390/biomedicines14102394/s1, Figure S1: Assessment of the estrous cycle by vaginal smear cytology; Figure S2: Characterization of rat bone marrow-derived mesenchymal stromal cells (rBM-MSCs); Figure S3. Peri-ovarian administration of free and alginate-encapsulated mitochondria.

## Abbreviations

rBM-MSC, rat bone marrow-derived mesenchymal stromal cell; MSC-Mito, free MSC-derived mitochondria; MSC-Mito@Alg, alginate-encapsulated MSC-derived mitochondria; SHE, sucrose–HEPES–EGTA; ΔΨm, mitochondrial membrane potential.
