# Supplementary material for: Peri-Ovarian Mitochondrial Administration Improves Surrogate Markers of Ovarian Function in Aged Rats: Free Versus Alginate-Encapsulated Mesenchymal Stromal Cell-Derived Mitochondria
Source: Biomedicines. 2026 Oct 20;14(10):2394. doi: 10.3390/biomedicines14102394 (PMC13701788; doi:10.3390/biomedicines14102394)
Supplement: Supplementary file 1 [file biomedicines-14-02394-s001.zip › biomedicines-4570765-supplementary.pdf]

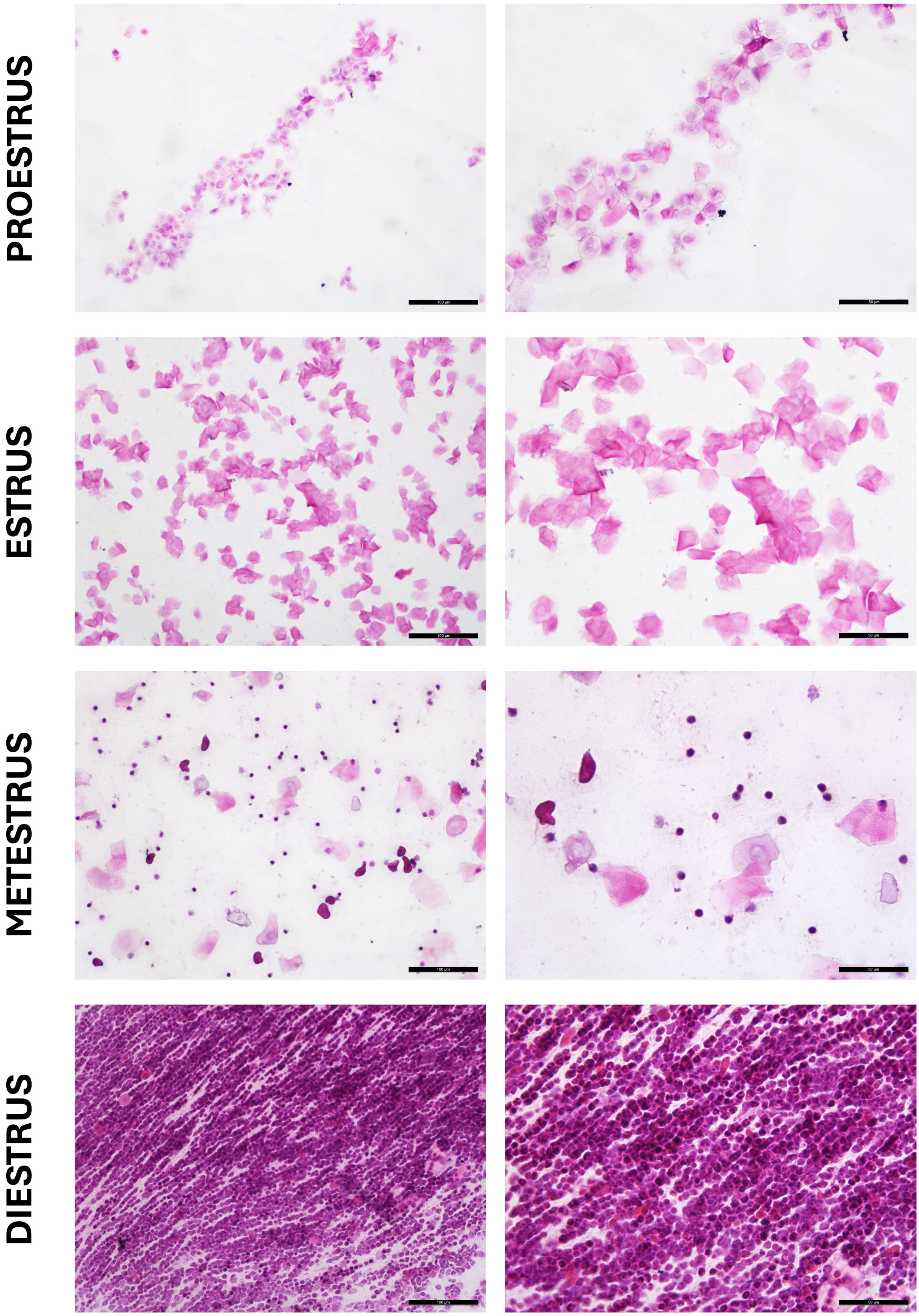

Figure S1. Assessment of the estrous cycle by vaginal smear cytology.

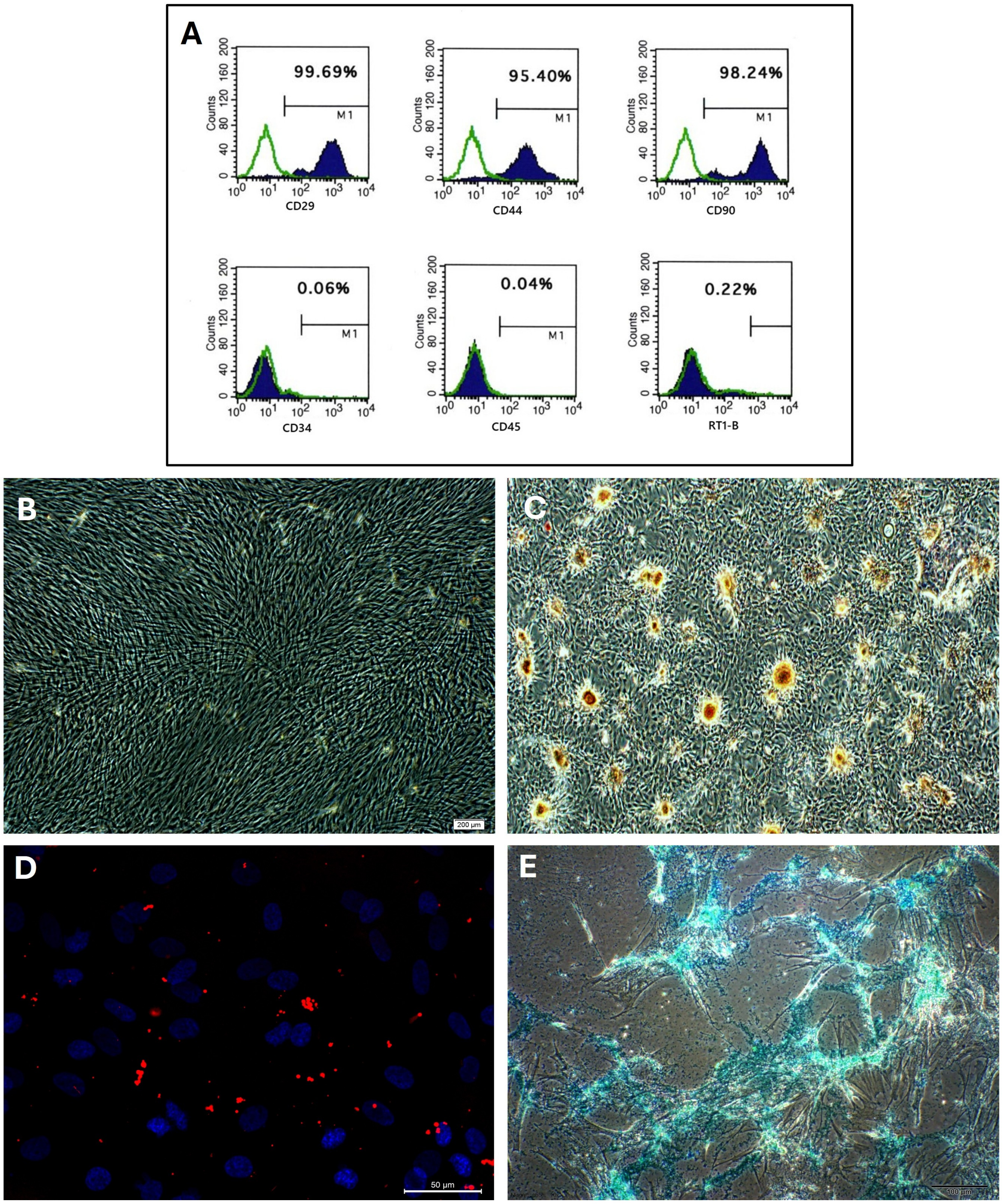

Figure S2. Characterization of rat bone marrow-derived mesenchymal stromal cells (rBM-MSCs).

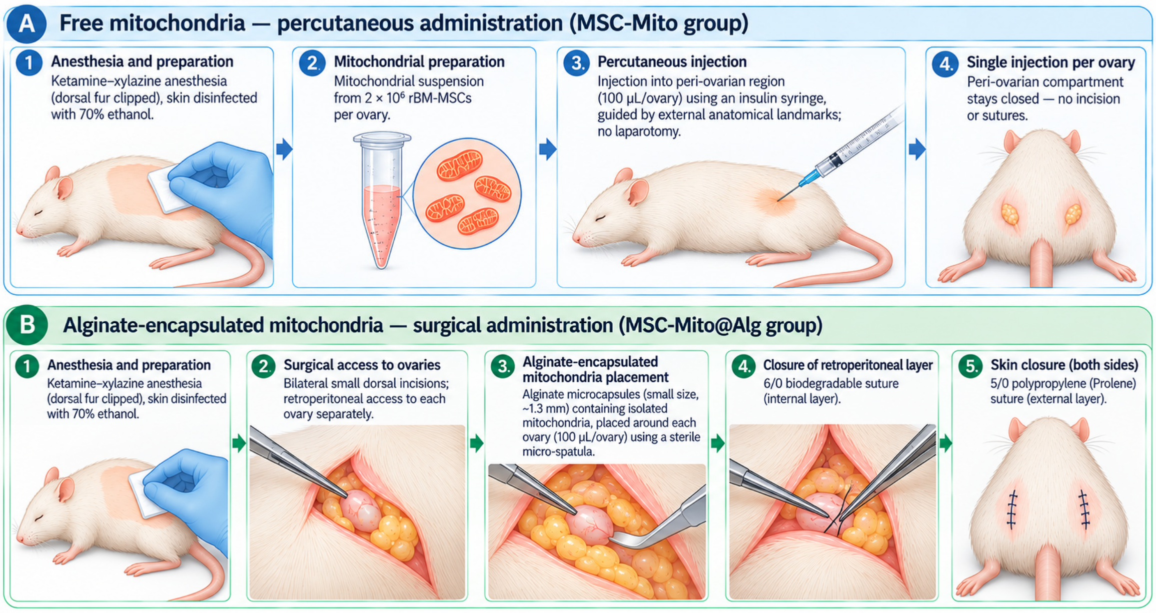

Figure S3. Peri-ovarian administration of free and alginate-encapsulated mitochondria. **(A)** Free mitochondria isolated from rat bone marrow-derived mesenchymal stromal cells (rBM-MSCs) were administered percutaneously into the peri-ovarian region (100 µL/ovary) without laparotomy. **(B)** Alginate microcapsules containing isolated mitochondria were surgically placed around each ovary through bilateral dorsal incisions and retroperitoneal access, followed by closure of the retroperitoneal layer and skin.
